# Coumarin-Based Prodrugs: Therapeutic Promise or Still Confined to Preclinical Exploration?

**DOI:** 10.3390/pharmaceutics18030341

**Published:** 2026-03-10

**Authors:** Atziri Corin Chavez Alvarez, Emmanuel Moreau

**Affiliations:** UMR-1240 Inserm, Université Clermont Auvergne, 58 Rue Montalembert, 63005 Clermont-Ferrand, France

**Keywords:** coumarins, prodrugs, drug delivery, medicinal chemistry, enzyme-responsive systems, multitarget therapy

## Abstract

Coumarin-based compounds are recognized for their chemical versatility and diverse biological activities, yet clinical applications remain largely confined to 4-hydroxycoumarin anticoagulants. To bridge this translational gap, coumarin scaffolds have been increasingly employed in prodrug design to enable controlled activation, targeted delivery, and theranostic functionality. This review critically evaluates whether coumarin-based prodrugs fulfill their therapeutic promise or remain primarily preclinical tools across oncology, inflammation, infectious diseases, and cardiovascular disorders. Strategies including enzymatic-, pH-, redox-, and light-triggered activation, as well as subcellular targeting and multifunctional hybrids, are discussed. Preclinical studies demonstrate improved bioavailability, reduced off-target toxicity, and real-time fluorescence monitoring, yet most compounds remain at the in vitro or small-animal model stage. Despite their mechanistic and conceptual potential, clinical translation is constrained by molecular complexity, pharmacokinetics, safety, and regulatory challenges. Overall, coumarins constitute a versatile multifunctional platform whose therapeutic impact relies on rigorous in vivo validation and strategic optimization.

## 1. Introduction

**Figure 1:**
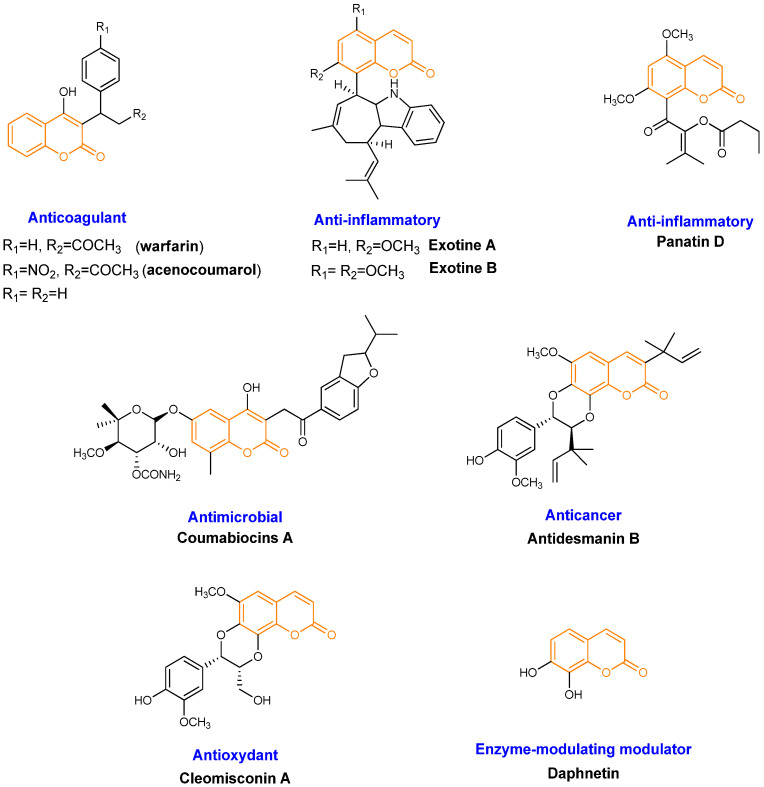
Molecular structures of different coumarin-derived compounds with different biological activities. In orange: the coumarinic moiety.

Coumarin-based compounds, commonly referred to as coumarins, represent an important class of naturally occurring and synthetically accessible molecules that have attracted significant attention in medicinal chemistry [1]. Structurally, coumarins are characterized by a benzopyrone core and are widely distributed in plants (Figure 1) [2,3,4]. Owing to their structural diversity, coumarins have been associated with a broad spectrum of reported biological activities, including anticoagulant, anti-inflammatory, antimicrobial, anticancer, antioxidant, and enzyme-modulating effects, which have positioned them as versatile scaffolds for drug discovery [5].

**Figure 2:**
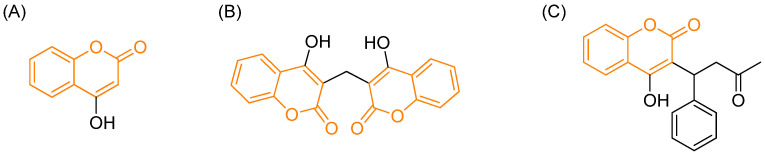
Molecular structure of (**A**) 4-hydroxycoumarin, (**B**) dicoumarol and (**C**) warfarin. In orange: the coumarinic moiety [9,10].

Despite this apparent pharmacological versatility and the abundance of data reported over several decades, the clinical translation of coumarin-based compounds has remained remarkably limited. In the early 20th century, a hemorrhagic syndrome in cattle fed spoiled sweet clover hay, the so-called “sweet-clover disease”, was traced to dicoumarol, a dimer of 4-hydroxycoumarin (Figure 2A,B). Dicoumarol was subsequently identified as a vitamin K epoxide reductase inhibitor, marking one of the first therapeutic applications of a coumarin derivative [6,7,8].

The structural simplicity and biological relevance of Dicoumarol inspired medicinal chemists to explore 4-hydroxycoumarin derivatives. Subsequent efforts led to the development of Warfarin and Acenocoumarol, two widely used oral anticoagulants, based on modifications of the 4-hydroxycoumarin core (Figure 2C) [10]. Early structure–activity relationship studies demonstrated that substituents on the coumarin ring (for example, at position C–8) significantly modulate anticoagulant potency [11].

To date, however, only a very limited number of coumarin-derived molecules have reached clinical use, essentially restricted to 4-hydroxycoumarin derivatives such as Warfarin and Acenocoumarol (Figure 1). These two compounds act as pharmacologically active agents, and their therapeutic relevance is confined to anticoagulation, with no approved applications in oncology, inflammatory, infectious, cardiovascular (beyond coagulation), or hepatic diseases [12,13,14,15].

This limited clinical success indicates that, in most cases, the coumarin scaffold does not possess sufficient intrinsic pharmacological potency or selectivity to support direct therapeutic development. Nevertheless, the lack of intrinsic clinical activity does not preclude the relevance of coumarins in drug design but rather shifts their role from active pharmacophores to functional molecular scaffolds.

**Figure 3:**
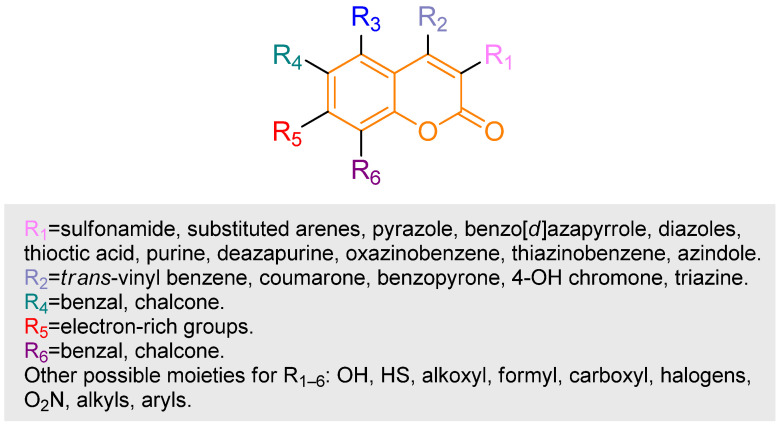
Substitutable positions and the possible moieties that can be used in each position that have previously demonstrated a biological effect Based on and adapted from Zeki et al., 2024 [19].

Since the 1990s, coumarins have been deliberately designed and investigated as prodrugs, a strategy aimed at improving the pharmacokinetic and pharmacodynamic profiles of active drugs by employing coumarin derivatives as carriers or precursors [16]. A prodrug is a molecule designed to exhibit minimal intrinsic activity until it is transformed by biological or chemical mechanisms in the body into its active pharmaceutical counterpart [17]. The interest in coumarin scaffolds for prodrug design stems from their well-defined chemical reactivity, modular structure, and predictable cleavage pathways under specific biological conditions [18]. From a drug delivery and biopharmaceutical standpoint, the coumarin core can offer considerable structural versatility, enabling fine control over chemical stability through rational substitution patterns and linker engineering (Figure 3). This versatility supports the design of prodrug systems that remain stable under physiological conditions while being selectively activated in response to specific biological triggers, thereby improving solubility, controlling drug release profiles, enhancing cellular uptake, and enabling selective activation in targeted biological environments.

Based on these design principles, coumarin-based prodrugs have been reported across a wide range of therapeutic areas. In the literature, these systems have been primarily investigated in the context of cancer, cardiovascular, inflammatory, metabolic and liver, neurodegenerative, and infectious diseases, reflecting both the chemical adaptability of the coumarin scaffold and the diversity of pathological environments targeted for prodrug activation.

In this context, this review provides a systematic and pathology-oriented analysis required to critically assess whether coumarin-based prodrugs represent viable therapeutic strategies or whether they primarily serve as proof-of-concept tools for controlled drug release and mechanistic exploration. By structuring the discussion around major disease areas such as cancer, cardiovascular, inflammatory, metabolic and liver, neurodegenerative, and infectious diseases, the review aims to critically examine the current state of the field and highlight opportunities and challenges for future development.

## 2. Coumarin-Based Prodrugs: Applications by Disease

### 2.1. Cancer

Cancer represents one of the most extensively investigated therapeutic areas in which coumarin-based prodrug strategies have been explored. This interest is driven not only by the inherent versatility of the coumarin scaffold but also by the diversity of pathological features associated with the tumor microenvironment, such as altered redox balance, dysregulated enzyme expression, and abnormal intracellular pH, that are particularly amenable to stimulus-responsive prodrug activation. From a prodrug design perspective, coumarin scaffolds serve as functional promoieties rather than as active pharmacophores. Their tunable lipophilicity and chemical robustness make them attractive for improving the delivery of established chemotherapeutic agents [20]. By conjugating anticancer drugs to coumarin backbones through ester, carbonate, or self-immolative linkers, reactive functional groups can be temporarily masked, potentially enhancing cellular uptake and enabling controlled intracellular release. In many reported systems, drug liberation relies on tumor-associated triggers such as esterases, β-glucuronidases, or glutathione-responsive reductases, providing a rationale for tumor-selective activation while aiming to limit systemic toxicity [12,16].

#### 2.1.1. Rationale for Using Coumarins in Prodrug Design for Cancer

**Figure 4:**
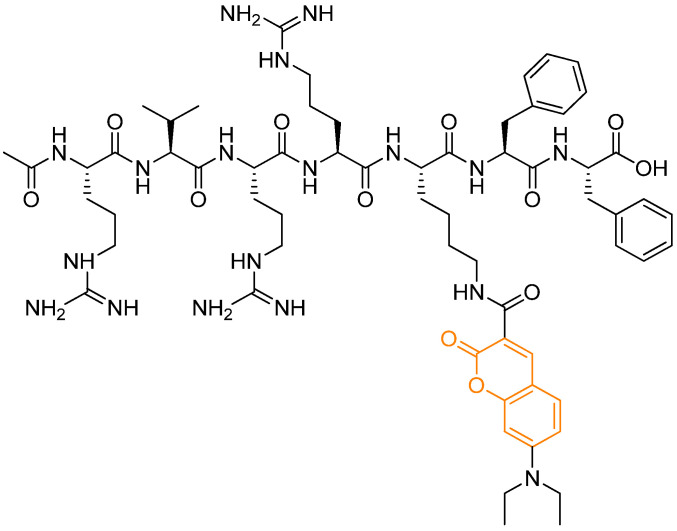
Self-assembling fluorescent probe known as RF-Cou containing 7-(diethylamino)-2-oxo-2H-chromene-3-carboxylic acid and a furin-responsive peptide. In orange: the coumarinic moiety. Based on and adapted from Chen et al., 2025 [25].

A particularly noteworthy aspect of coumarins in cancer therapy is their photophysical behavior. Many coumarin derivatives serve as excellent chromophores, displaying strong fluorescence, high stability, and tunable excitation wavelengths [15,21]. These properties enable the design of theranostic prodrugs, which integrate diagnostic imaging with therapeutic action [22]. Such coumarin-based theranostic agents allow real-time visualization of drug distribution, intracellular trafficking, and activation pathways [22,23]. Some coumarin prodrugs are engineered to remain inactive until exposed to specific wavelengths of light, enabling photoactivated chemotherapy that precisely controls when and where cytotoxic agents are released. In representative systems, a lead compound displays a “dark” cytotoxicity of 116 nM, which is restored upon irradiation to a photoactivated activity of 13 nM [24]. This strategy minimizes damage to healthy tissue and enhances the therapeutic index of potent anticancer drugs. Moreover, the use of coumarin fluorophores facilitates quantitative monitoring of drug activation kinetics within tumors, providing valuable information for dosage optimization and personalized treatment planning [14]. In recent years, advances in coumarin photochemistry have further expanded their utility in systems such as light-triggered self-immolative cascades (Figure 4), fluorescent apoptosis trackers, and tumor microenvironment-responsive probes [25,26].

One illustrative example is the development of mutual prodrugs linking coumarins to established chemotherapeutic agents such as 5-fluorouracil (5-FU). In these systems, the hydroxyl group of a coumarin derivative is esterified with 5-FU or with functional analogs such as dichloroacetic acid to form a conjugate that remains stable at physiological pH but undergoes enzymatic cleavage in serum [27,28]. This approach improves cellular uptake and reduces the gastrointestinal toxicity traditionally associated with 5-FU, while the coumarin fragment may contribute additional antiproliferative activity. Another notable example is the design of pH-responsive doxorubicin–coumarin conjugates, which exploit the acidic tumor microenvironment to trigger selective hydrolysis of the linker and release doxorubicin preferentially in cancerous tissues [29]. In these systems, drug release is more pronounced under acidic conditions (pH 5.0) than at physiological pH (7.4). In many such designs, coumarin fluorescence is initially quenched and restored upon cleavage, enabling visualization of intracellular drug release.

**Figure 5:**
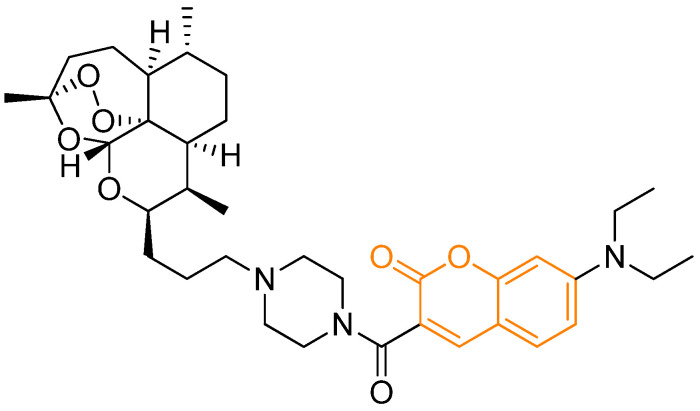
Structure of 7-(diethylamino)-3-(4-(3-((3*R*,5a*S*,6*R*,8a*S*,9*R*,10*R*,12*R*,12aR)-3,6,9-trimethyldecahydro-3H-3,12-epoxy [1,2]dioxepino [4,3-i]isochromen-10-yl)propyl)piperazine-1-carbonyl)-2H-chromen-2-one, an artemisin–coumarin conjugate prodrug. In orange: the coumarinic moiety. Based on and adapted from Zhang et al., 2015 [22].

More complex strategies involve light-activated coumarin prodrugs, in which the drug remains inactive until irradiation with visible or near-infrared light [24]. In representative systems, a coumarin moiety is attached to a cytotoxic agent through a photocleavable linker; following cellular accumulation, controlled light exposure releases the active drug and simultaneously restores coumarin fluorescence. This dual-activation mechanism enables precise spatiotemporal control of chemotherapy while minimizing off-target toxicity. Additional sophistication is achieved in enzyme-responsive nanosystems where coumarin acts as both a reporter and a structural component of a pro-prodrug [30]. Another important example is the development of artemisinin–coumarin conjugates aimed at targeting cancer cell mitochondria (Figure 5) [22].

In these hybrids, the coumarin fragment drives preferential accumulation in mitochondria due to its lipophilicity and compatibility with mitochondrial membranes, while artemisinin contributes potent ROS-mediated cytotoxicity. Artemisinin–coumarin conjugates display cytotoxic activity in the low micromolar range (3–24 μM) in hepatic cancer (HepG2, Hep3B) and ovarian cancer (A2780, OVCAR-3) cell lines, whereas artemisinin alone remains inactive at concentrations up to 100 μM [22]. The intrinsic fluorescence of the coumarin moiety enables live-cell imaging, rendering these constructs genuine theranostic agents capable of reporting their own intracellular localization. Targeted prodrugs have also been developed by combining coumarins with recognition motifs such as biotin. For instance, gemcitabine–coumarin–biotin conjugates linked via a disulfide bond exploit elevated intracellular glutathione levels to achieve selective drug release in tumor cells [23]. Fluorescence activation provides a real-time readout of drug liberation, while biotin enhances cellular uptake through overexpressed biotin receptors.

Across these examples, coumarin derivatives emerge as multifunctional platforms capable of improving drug stability, enhancing tumor selectivity, enabling triggered activation, and providing valuable imaging capabilities. While coumarins do not exert direct anticancer activity, they play a crucial role in enhancing the efficacy of cytotoxic agents by acting as intelligent sensing units for real-time monitoring or as chemical shields that improve stability, loading capacity, and bioavailability.

While these examples highlight the conceptual advantages of coumarins as promoieties in anticancer prodrug design, their functional potential, particularly regarding activation efficiency and selectivity, can be assessed through the in vitro studies discussed in the following section.

#### 2.1.2. Mechanisms of Activation and In Vitro Performance

The exploitation of redox-sensitive mechanisms and membrane-targeting strategies represents one of the earliest and most prominent approaches in the development of coumarin-based prodrugs for cancer therapy. For example, theranostic conjugates combining gemcitabine (GMC), a coumarin moiety, and biotin have been developed to selectively target biotin receptors overexpressed in certain cancer cell lines. In this system, the coumarin unit acts as a fluorescent reporter, with its emission intensity increasing by approximately 3.5-fold following intracellular cleavage of a disulfide linker triggered by glutathione (GSH) [23]. In vitro studies conducted on A549 lung adenocarcinoma cells demonstrated the effectiveness of this targeting strategy: at a concentration of 1.0 μM, the biotin-targeted prodrug reduced cell viability to approximately 15%, compared with around 50% for the non-biotinylated analog.

Subcellular targeting of mitochondria represents another significant strategy to enhance the activity of natural products with otherwise limited anticancer potency. Recent studies have shown that the use of a coumarin-3-carboximide fluorophore enables selective delivery of artemisinin to the mitochondria of hepatocellular carcinoma cells (HepG2 and Hep3B) [22]. While native artemisinin and the coumarin moiety alone exhibit negligible cytotoxicity (IC_50_ > 100 μM), the resulting theranostic conjugate achieves an IC_50_ value of 3.05 μM in HepG2 cells. Importantly, this system displays high selectivity, with an IC_50_ of 104.17 μM in normal IOSE-144 cells, highlighting the critical role of mitochondrial targeting in triggering apoptosis through localized overproduction of reactive oxygen species (ROS).

Spatiotemporal control through visible-light photoactivation provides an additional level of precision to limit systemic side effects. “Double-activation” systems have been designed that require both intracellular esterase activity and irradiation at 540 nm to release cytotoxic agents such as combretastatin A-4 (CA-4) [24]. This prodrug architecture reduces dark toxicity by a factor of 14.5. Upon irradiation (8 mW·cm^−2^ for 30 min), approximately 99% of the active agent is released, restoring cytotoxicity comparable to that of the parent drug (IC_50_ = 13 nM for the activated prodrug versus 8 nM for free CA-:4).

Structural optimization and kinetic control of drug release are essential parameters for fine-tuning therapeutic responses. Early work by [16] demonstrated that prodrug structure dramatically influences esterase-mediated release rates, with half-lives ranging from 2 to 190 min depending on the nature of the delivered amine (Wang et al., 1998). Amines with lower pKₐ values, corresponding to better leaving groups, were released more rapidly, as exemplified by the p-anisidine prodrug (t_1_/_2_ ≈ 2 min). In the context of photoactivation, ref. [26] achieved a major technological advance by stabilizing the intermediate chromophore cation through allylic substitution at the α-carbon of the coumarin scaffold. This molecular engineering strategy increased the photolysis quantum yield by up to 35-fold, enabling near-instantaneous release of complex payloads such as piperacillin. Notably, the antibacterial activity against *E. coli* (minimal inhibitory concentration (MIC) = 0.66–0.88 μM) could be accurately predicted based on the fluorescence signal generated during drug release.

Finally, enzyme-specific activation by tumor-associated proteases such as furin has been exploited for high-precision imaging applications. A peptide-based probe conjugated to a coumarin fluorophore (RF-Cou) was shown to undergo enzyme-triggered self-assembly into nanoparticles following proteolytic cleavage, inducing a transition from monomer to excimer fluorescence [25]. In murine 4T1 breast cancer cells, the fluorescence intensity in the excimer emission channel (550 nm) became approximately 3.2-fold higher than that of the monomer channel after 6 h of incubation, providing high-contrast molecular imaging of protease activity.

Overall, these in vitro studies confirm that the coumarin scaffold represents a highly versatile functional platform in oncology. Beyond masking the toxicity of potent chemotherapeutic agents, coumarins can act as targeting vectors and fluorescent sentinels, enabling real-time monitoring of drug delivery and activation at the subcellular level, particularly within lysosomes and mitochondria.

Despite the wealth of in vitro data supporting the use of coumarin scaffolds as versatile prodrug platforms, most reported systems remain confined to cellular models. The translational potential of these approaches can only be established through in vivo studies addressing key parameters such as stability, biodistribution, tumor selectivity, and therapeutic benefit. The next section therefore examines coumarin-based prodrugs that have been evaluated in vivo in cancer models, highlighting both their successes and the challenges that currently limit their progression toward clinical application.

Overall, these in vitro studies confirm that the coumarin scaffold represents a highly versatile functional platform in oncology. Beyond masking the toxicity of potent chemotherapeutic agents, coumarins can act simultaneously as targeting vectors and fluorescent sentinels, enabling real-time monitoring of drug delivery and activation at the subcellular level, particularly within lysosomes and mitochondria. The integration of stimulus-responsive linkers further allows precise spatial and temporal control over drug release, contributing to enhanced selectivity and reduced off-target effects. Despite the wealth of in vitro data supporting coumarin-based systems as multifunctional prodrug platforms, most reported examples remain confined to cellular models. Their true translational potential can only be assessed through in vivo studies addressing key parameters such as stability, biodistribution, tumor selectivity, and therapeutic benefit.

#### 2.1.3. Cancer-Related In Vivo Studies of Coumarin-Based Prodrugs

The integration of coumarin motifs into the design of theranostic prodrugs represents a major step forward in targeted cancer therapy. Owing to its intrinsic fluorescence and structural adaptability, coumarin enables stimulus-responsive drug release while allowing real-time monitoring of therapeutic outcomes in complex tumor environments.

The use of coumarin as a photocleavable molecular “lock” has proven particularly effective when combined with light-penetration technologies. For instance, Brion et al. (2023) [31] developed lipid nanocapsules containing a coumarin linker (DEACAS) to mask melphalan activity. Upon activation by deep red light (641 nm) through upconversion, this system markedly suppressed tumor growth in MDA-MB-231 murine models, outperforming the free drug at equivalent doses. In another example, Ni et al. (2026) [32] reported the intratumoral formation of a two-photon-activatable photosensitizer, where coumarin served as an electron donor. Activated by endogenously overexpressed sulfite in H22 tumors, the system achieved nearly complete tumor ablation under 800 nm irradiation, remaining effective even under hypoxic conditions. Similarly, Huang et al. (2025) [33] exploited coumarin as a thiol (Cys/GSH)-responsive recognition site to activate a photodynamic therapy (PDT) probe in 4T1 tumors, yielding pronounced tumor necrosis and confirming coumarin’s dual therapeutic and diagnostic (theranostic) role. In summary, coumarin-activated phototherapy enables precise spatial and temporal control of anticancer activity while providing real-time, in vivo fluorescence tracking of therapeutic response.

Beyond photochemical activation, coumarin-based systems can achieve selectivity through bioorthogonal chemistry or subcellular localization. Li et al. (2017) [34] developed a palladium-activated nitrogen mustard prodrug that achieved a 67.1% tumor inhibition rate in HeLa xenografts while minimizing the systemic toxicity typical of the unmodified drug. Han et al. (2021) [35] further demonstrated mitochondrial targeting by conjugating a coumarin derivative to dihydroartemisinin (DHA). This nanosystem enabled fluorescent visualization of drug release and cytochrome c-mediated apoptosis. In MDA-MB-231-bearing mice, the treatment achieved 68.9% tumor suppression, highlighting the benefits of combining selective localization with real-time imaging. Overall, bioorthogonal activation and mitochondrial targeting illustrate coumarin’s capacity to guide prodrugs to precise subcellular environments while ensuring direct visualization of therapeutic processes.

Finally, coumarin also contributes to formulation stability and pharmacological performance. Xu et al. (2015) [36] utilized π–π interactions between coumarin moieties and SN-38 to enhance solubility and intratumoral retention. In an HT-29 colon cancer model, this coumarin-based nanoplatform surpassed irinotecan, the standard clinical prodrug, by extending median survival from 35 to 44 days. Thus, coumarin not only stabilizes nanocarrier formulations but also enhances bioavailability and overall therapeutic benefit, reinforcing its multifunctional value in advanced oncologic prodrugs.

#### 2.1.4. Global Conclusion on Cancer

Cancer represents one of the most extensively studied therapeutic areas for coumarin-based prodrug strategies. This interest arises from both the versatility of the coumarin scaffold and the distinctive characteristics of the tumor microenvironment, including altered redox balance, dysregulated enzyme expression, and abnormal intracellular pH, which can be exploited to trigger stimulus-responsive prodrug activation. In these systems, coumarins mainly act as functional promoieties rather than active pharmacophores. Their tunable lipophilicity and chemical stability make them suitable for improving the delivery of established chemotherapeutic agents. By linking anticancer drugs to coumarin frameworks through ester, carbonate, or self-immolative linkers, reactive functional groups can be temporarily masked, facilitating cellular uptake and enabling controlled intracellular release. Overall, these properties highlight the potential of coumarin-based prodrugs as multifunctional platforms in oncology, capable of enhancing drug delivery, enabling tumor-responsive activation, and, due to their intrinsic fluorescence, allowing real-time monitoring of drug release and intracellular distribution.

### 2.2. Inflammatory Diseases and Pain

#### 2.2.1. Background and Rationale

Coumarin-based prodrugs and derivatives have attracted substantial interest in the treatment of inflammatory diseases and pain due to their ability to reduce adverse effects, enhance bioavailability, and introduce multifunctional behavior into classical anti-inflammatory agents.

Several studies demonstrated that coumarins possess intrinsic anti-inflammatory activity, which makes them particularly valuable as scaffolds for prodrug design [37,38]. Beyond direct anti-inflammatory effects, coumarin frameworks have been used to design prodrugs that deliver classical NSAIDs through enzymatically cleavable linkers [39]. Masking the carboxyl group of NSAIDs as esters is a well-established prodrug strategy that increases lipophilicity and membrane permeability and can reduce direct gastric irritation [40,41]. In related approaches, aromatic phenolic promoieties, including hydroxylated heterocycles such as coumarins, have been used to form ester or mutual prodrug conjugates that improve absorption and act as releasable carriers [42,43].

Collectively, these findings highlight coumarin derivatives and coumarin-based prodrugs as highly versatile agents in the treatment of inflammation and pain. Their ability to modulate cytokine signaling, inhibit pro-inflammatory enzymes, scavenge reactive oxygen species, deliver NSAIDs with reduced toxicity, and interact with nociceptive pathways positions coumarins as a powerful platform for next-generation anti-inflammatory drug discovery. As interest in polypharmacology and targeted prodrug activation continues to grow, coumarins stand out as one of the most promising scaffolds for developing safer and more effective therapies for both acute and chronic inflammatory diseases.

#### 2.2.2. Mutual Prodrugs for Gastrointestinal Protection: The Flurbiprofen–Umbelliferone Model

The incorporation of the coumarin nucleus (2H-1-benzopyran-2-one) into prodrug design has been widely explored to improve the therapeutic index of non-steroidal anti-inflammatory drugs (NSAIDs), particularly by mitigating gastrointestinal toxicity. In this context, Ashraf et al. (2016) [42] developed a series of mutual prodrugs by esterifying the free carboxylic acid of flurbiprofen with natural antioxidant moieties, including umbelliferone (7-hydroxycoumarin) as a coumarin-based promoiety. The resulting coumarin–NSAID conjugate (compound **4b**) exhibited high chemical stability in simulated gastric fluid (pH 1.2), thereby limiting direct gastric irritation. Enzymatic cleavage by plasma esterases enabled efficient regeneration of the active drug, with release rates ranging from 61% to 92%. Pharmacological evaluation demonstrated that compound 4b surpassed native flurbiprofen in analgesic efficacy (75% versus 69% inhibition), while markedly reducing the ulcerogenic index from 3.07 (flurbiprofen) to 1.34 (prodrug). These results illustrate how coumarin promoieties can be leveraged to improve tolerability without compromising anti-inflammatory efficacy.

#### 2.2.3. Theranostic Systems for Site-Specific Activation: The Azo-Coumarin Approach

More recent strategies have extended the role of coumarins beyond simple promoieties toward theranostic prodrug systems that combine drug release with real-time monitoring. Wang et al. (2025) [44] designed a colon-targeted prodrug (P1) for the treatment of ulcerative colitis by linking 5-aminosalicylic acid (5-ASA) to the fluorescent reporter 7-amino-4-methylcoumarin (7-AMC) via an azo bond. In this system, fluorescence of the coumarin moiety is quenched by the azo linker (FRET effect). Upon reaching the colon, bacterial azoreductases selectively cleave the azo bond, simultaneously releasing 5-ASA and restoring coumarin fluorescence. A 28-fold fluorescence enhancement was observed within 4 h, enabling precise visualization of drug activation. When encapsulated into polymeric micelles, this prodrug showed therapeutic efficacy comparable to free 5-ASA in restoring colonic mucosal integrity, while allowing ex vivo tracking of biodistribution. This example highlights how coumarins can function as dual therapeutic and imaging components in inflammation-targeted prodrug systems.

#### 2.2.4. Structural Optimization Versus True Prodrug Behavior: Esters of Coumarins

Not all coumarin esters necessarily operate through a classical prodrug mechanism. Al-Wabli et al. (2018) [45] synthesized a series of 7-substituted coumarin esters to increase lipophilicity and improve biological activity. Although these compounds are formally esters, in vitro assays (albumin denaturation and red blood cell membrane stabilization) suggested that the observed anti-inflammatory effects arose from the intact molecules rather than from metabolic hydrolysis to parent coumarins. Among this series, compound 6, bearing a 4-chlorobenzoyl substituent, exhibited the most pronounced activity, achieving 94.87% inhibition of edema in vivo, comparable to celecoxib (92.32%). This study underscores the importance of mechanistic validation when classifying coumarin derivatives as prodrugs and demonstrates that the coumarin scaffold itself can enhance affinity for inflammatory targets such as Cyclooxygenase (COX) enzymes without requiring enzymatic release.

#### 2.2.5. Prodrug Strategies to Improve Oral Bioavailability: N-Alkylated Dihydroisocoumarins

A classical yet highly instructive example of coumarin-based prodrug design is provided by Shimojima et al. (1985) [46], who addressed the poor oral absorption of a naturally occurring dihydroisocoumarin. The parent compound, AI-77-B (**1**), isolated from *Bacillus pumilus*, exhibits potent gastroprotective and anti-inflammatory activity but is completely inactive after oral administration due to negligible gastrointestinal absorption. To overcome this limitation, the authors designed lipophilic prodrugs through two key chemical modifications: (i) lactonization of the amino acid-derived side chain and (ii) *N*-alkylation of the primary amine with short alkyl chains (series **5a**–**f**). These transformations yielded γ-lactone derivatives capable of intestinal absorption. Following uptake, the prodrugs underwent NADPH-dependent enzymatic *N*-dealkylation in the liver, regenerating the active drug in systemic circulation. In vivo evaluation in rats demonstrated a dramatic restoration of oral efficacy. While AI-77-B (**1**) showed no gastroprotective activity (0% protection against stress-induced ulcers at 50 mg/kg, p.o.), compound **5c** (n-propyl) afforded 94% protection, and compound **5b** (ethyl) afforded 78% protection. Pharmacokinetic studies further confirmed effective absorption, with peak blood concentrations of 38.5 µg/mL (**5b**) and 22.2 µg/mL (**5c**) observed 1 h after oral dosing (200 mg/kg), whereas the parent compound remained undetectable. Compound **5c** also showed oral anti-inflammatory activity with ED_50_ values of 124 mg/kg (carrageenan-induced edema) and 69 mg/kg (bradykinin-induced edema). Acute toxicity was low, with oral LD_50_ values of 1250 mg/kg (**5b**) and 1075 mg/kg (**5c**). This study demonstrates that *N*-alkylated γ-lactone prodrugs can convert poorly absorbed coumarin-derived natural products into orally active anti-inflammatory agents through enhanced absorption and metabolic activation.

#### 2.2.6. Global Conclusion on Inflammatory Diseases and Pain

Taken together, these studies illustrate how coumarins can fulfill multiple roles in anti-inflammatory drug design, ranging from intrinsic bioactive scaffolds to promoieties enabling controlled drug release, site-specific activation, or improved pharmacokinetic profiles. As observed in oncology, most advances remain supported by in vitro and early in vivo data and should be regarded as proof-of-concept demonstrations rather than evidence of clinical translation. Nevertheless, the chemical versatility of coumarins and their compatibility with diverse activation mechanisms position them as valuable tools for the rational design of next-generation anti-inflammatory prodrugs.

### 2.3. Infectious Diseases

#### 2.3.1. Background and Rationale

**Figure 6:**
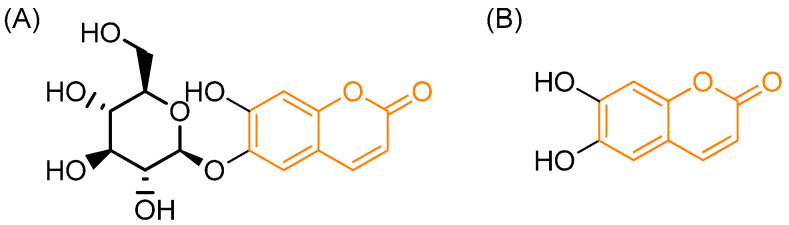
Molecular structure of (**A**) the prodrug esculin and (**B**) its active metabolite esculetin. In orange: the coumarinic moiety. Based on and adapted from Zaragozá et al., 2021 [49].

Several studies have demonstrated that coumarins possess intrinsic antimicrobial activity, which makes them particularly valuable as scaffolds for prodrug design. In antibacterial applications, coumarins and their derivatives can serve as carriers that mask polar functionalities of conventional antibiotics, improving membrane permeability and cellular uptake. Once inside microbial or host cells, enzymatic or chemical cleavage liberates the active drug, while the coumarin moiety may itself exert antibacterial effects by interfering with DNA gyrase or topoisomerase IV, disrupting microbial replication with inhibitory concentrations as low as 0.05 mg/L [47]. For fungal infections, natural coumarin glycosides such as esculin illustrate a classical prodrug model: esculin is hydrolyzed by microbial or host β-glucosidases to yield esculetin, the active antifungal agent (Figure 6) that exhibits a minimal inhibitory concentration of 22 µg/mL against organisms such as *Trichophyton soudanense* versus esculin, which remains inactive (>3402.8 µg/mL) [48]. Inspired by this, synthetic coumarin prodrugs have been developed that exploit fungal or host enzyme expression to selectively release antifungal drugs in infected tissues, thereby reducing systemic toxicity and improving selectivity.

In settings where viruses pose a risk, coumarin-based prodrugs offer a flexible platform to deliver antiviral agents or bioactive coumarin fragments that may interfere with viral replication, entry, or protein processing [50,51]. Moreover, coumarin’s favorable pharmacokinetics and metabolic stability make it a useful vector for delivering antimicrobial agents to tissues with poor drug penetration, such as biofilm-associated infections or fungal reservoirs [52]. Coumarins are used either as a structural scaffold to create potent hybrids against resistant bacteria and viruses or as prodrugs that exploit their solubility to improve treatment delivery. Their ability to be chemically modified while maintaining low systemic toxicity makes them particularly attractive candidates for the development of multi-target drugs capable of simultaneously addressing infection, inflammation, and oxidative stress.

#### 2.3.2. In Vitro Studies: Enzyme-Targeted and Glycosylated Coumarin Prodrugs

In bacterial carbonic anhydrases, Giovannuzzi et al. (2022) [53] demonstrated that coumarins act as suicide inhibitors against α-carbonic anhydrases from pathogens such as *Neisseria gonorrhoeae* (NgCAα) and Vibrio cholerae (VchCAα). The activation mechanism is intrinsic to the enzyme: the esterase activity of α-CAs hydrolyzes the coumarin lactone ring to generate 2-hydroxycinnamic acids, which subsequently bind at the enzyme active site and block catalytic activity. The substituted coumarins exhibited inhibition constants ranging from 28.6 to 469.5 µM for NgCAα and 39.8 to 438.7 µM for VchCAα, with 4-methyl-7-diethylaminocoumarin (compound **8**) being the most potent against NgCAα (KI = 28.6 µM). A notable advantage of this approach is its selectivity, as the coumarins showed minimal activity against human isoforms hCA I and II, reducing off-target effects. For superficial fungal infections, Mercer et al. (2013) [48] explored glycosylated coumarins as prodrugs to overcome the low aqueous solubility of natural antifungals while ensuring local drug release. Esculin (6-glucoside) is highly water-soluble (~6 g/L) and biologically inactive until hydrolyzed by β-glucosidases secreted by dermatophytes such as *Trichophyton* rubrum, releasing the aglycone esculetin (6,7-dihydroxycoumarin), which demonstrates antifungal activity with MIC values of 174.18 µg/mL against *T. interdigitale* and 348.36 µg/mL against *T. rubrum*. The effect is abolished in the presence of conduritol B epoxide, a β-glucosidase inhibitor, confirming enzyme-mediated activation.

Shimojima et al. (1985) [46] illustrated a classical oral prodrug strategy to enhance the bioavailability of poorly absorbed natural products using AI-77-B, a dihydroisocoumarine with gastroprotective and anti-inflammatory properties. Because AI-77-B itself is inactive orally due to negligible intestinal absorption, derivatives were designed with increased lipophilicity via lactonization of the amino acid side chain and *N*-alkylation of the primary amine (derivatives **5a**–**f**). These lipophilic prodrugs are absorbed in the small intestine and subsequently undergo NADPH-dependent enzymatic dealkylation in the liver to regenerate the active compound. In rats, oral administration of the native drug provided no gastroprotective activity, while derivatives **5b** (ethyl) and **5c** (n-propyl) yielded 78% and 94% protection against stress-induced ulcers at 50 mg/kg, respectively. Blood concentrations reached 38.5 µg/mL for **5b** and 22.2 µg/mL for **5c** one hour post-administration (200 mg/kg, p.o.). The anti-inflammatory ED_50_ values of **5c** were 124 mg/kg for carrageenan-induced paw edema and 69 mg/kg for bradykinin-induced edema, with low acute toxicity (LD_50_: **5b** = 1250 mg/kg; **5c** = 1075 mg/kg).

Collectively, these in vitro studies highlight the versatility of the coumarin scaffold to generate active antimicrobial agents locally via enzyme-triggered activation and to improve oral bioavailability of poorly absorbed compounds through rational prodrug design.

#### 2.3.3. In Vivo Studies: Multi-Target and Antiviral Applications

Alshibl et al. (2020) [54] demonstrated that hybrid coumarins, combining pyranocoumarin or sulfonamide motifs, exhibit remarkable multi-target activities. Sulfonamide derivatives **8c** and **8d** showed potent antioxidant activity with IC_50_ values of 3.87 and 4.30 µg/mL, while pyranocoumarin **5a** inhibited rat paw edema in vivo by 29.2% within the first hour and displayed high selectivity for COX-2 (SI = 152). Several coumarin-sulfonamide hybrids (**7c,d**; **8c,d**; **9c,d**) demonstrated antibacterial and antifungal activities comparable or superior to standard drugs such as ciprofloxacin and ketoconazole, with MIC values of 125 µg/mL. Beyond classical antibacterial effects, coumarin scaffolds have shown antiviral potential, as reviewed by Mishra et al. (2020) [50]. Certain diazocoumarins inhibit HIV replication at levels comparable to AZT, whereas coumarin–benzimidazole conjugates suppress up to 90% of HCV RNA replication. Other derivatives, such as eleutheroside B1, interfere with viral gene expression, showing promise against influenza and emerging viruses, including Dengue and Chikungunya. The chemical flexibility, metabolic stability, and favorable pharmacokinetics of coumarins support their use for multi-target antiviral applications in vivo.

#### 2.3.4. Global Conclusion on Anti-Infective Applications

The integration of enzyme-triggered prodrugs, glycosylated derivatives, hybrid scaffolds, and antiviral coumarins illustrates the remarkable versatility of the coumarin scaffold in anti-infective therapy. Coumarin-based prodrugs allow targeted activation in bacterial and fungal infections, improve oral bioavailability of poorly absorbed natural products, and enable multi-target effects encompassing antimicrobial, antiviral, anti-inflammatory, and antioxidant activities. In vitro and in vivo evaluations collectively support the development of selective, potent, and low-toxicity agents capable of addressing antibiotic resistance, fungal infections, and emerging viral threats, establishing coumarins as a robust platform for next-generation anti-infective therapeutics.

### 2.4. Cardiovascular Diseases

#### 2.4.1. Background and Rationale

Coumarin-based prodrugs have generated significant interest in cardiovascular medicine due to their ability to modulate thrombosis, oxidative stress, endothelial dysfunction, and lipid dysregulation through both direct biological activities and controlled drug release strategies [49]. Historically, the anticoagulant properties of 4-hydroxycoumarins laid the foundation for cardiovascular applications, but modern approaches extend far beyond classical vitamin K antagonists [55]. Newer coumarin derivatives have been engineered as prodrugs designed to release antithrombotic, vasoprotective, or cardiometabolic agents in a more targeted manner, thereby improving therapeutic windows and reducing adverse effects [56]. For example, ester-linked coumarin conjugates can mask acidic or highly polar groups from antiplatelet drugs, enhancing their oral absorption and facilitating timed release through enzymatic hydrolysis. These coumarinic compounds were designed to resist esterase activity, which was successfully obtained. The lead compounds exhibited half-lives of 95 and 84 min in porcine liver esterase [20]. Some coumarin prodrugs exploit the elevated esterase activity associated with inflamed or ischemic vascular tissue, allowing selective activation at sites of endothelial injury or plaque formation [57]. Additionally, coumarins possess intrinsic antioxidant, anti-inflammatory, and vasodilatory properties, such as inhibition of ROS production, attenuation of nitric oxide imbalance, and modulation of key enzymes involved in vascular tone. These complementary activities suggest that coupling cardiovascular drugs with coumarin scaffolds may yield dual-action molecules capable of addressing multiple pathological mechanisms at once, such as thrombosis, oxidative injury, and vascular inflammation, while maintaining favorable pharmacokinetic properties. As cardiovascular diseases increasingly demand multitargeted interventions, coumarin-derived prodrugs represent a promising avenue for achieving improved efficacy with reduced systemic toxicity.

#### 2.4.2. In Vitro Evaluation of Coumarin-Based Prodrugs in Cardiovascular Therapy

The use of the coumarin scaffold (2H-1-benzopyran-2-one) as a prodrug platform offers unique opportunities in cardiovascular drug design, particularly for improving oral bioavailability, selective enzyme targeting, and antiplatelet activity.

- *Enhancing Oral Absorption of Peptidomimetic RGD Analogs*

Wang et al. (2000) [20] addressed the low oral bioavailability of fibrinogen antagonists (RGD analogs) by designing cyclic coumarin-based prodrugs. The native compounds, such as tirofiban (MK-383), are highly hydrophilic and charged, limiting intestinal membrane permeation. By masking polar groups (carboxyl and amino) through cyclization, the resulting prodrugs exhibited significantly increased lipophilicity, as evidenced by IAM-HPLC measurements (log kw values increasing from 1.22 to 1.23 to 3.60–3.71). In Caco-2 cell models, these prodrugs showed 5–6 fold higher cellular permeability compared to their parent molecules. Oral administration of the tirofiban-derived prodrug 1c in dogs produced significant and prolonged antiplatelet activity, whereas the native drug was minimally active at equivalent doses. Bioconversion studies revealed esterase-mediated activation, with half-lives in human plasma of 91 min (**1a**) and 57 min (**1b**), demonstrating efficient in situ regeneration of the active compound.

- *Antiplatelet Activity and Mechanistic Insights*

Zaragozá et al. (2021) [49] investigated the antiaggregatory effects of coumarin, esculetin (aglycone), and esculin (glycoside) in vitro, focusing on COX-1 interactions. Coumarin and esculetin showed stronger inhibition of platelet aggregation than esculin. In whole blood activated by arachidonic acid, the IC_50_ values were 2.45 mM for coumarin and 3.07 mM for esculetin. Platelet-rich plasma experiments revealed complete inhibition at 1.5 mM for coumarin. Structure–activity analysis indicated that aglycones have superior platelet access compared to glycosylated forms. Esculetin inhibited COX-1 activity up to 90% at 6.5 mM, outperforming coumarin (60%), whereas esculin paradoxically showed the strongest inhibition on the recombinant h-COX-1 enzyme (74%, IC_50_ = 4.49 mM). Notably, none of the tested coumarins significantly affected thromboxane B2 levels, suggesting a mechanism possibly involving TXA2 receptor modulation rather than direct suppression of thromboxane synthesis.

#### 2.4.3. Global Conclusion on Cardiovascular Applications

Collectively, these in vitro studies demonstrate the versatility of the coumarin scaffold in cardiovascular drug design. Coumarin-based prodrugs can enhance oral bioavailability of charged peptidomimetics, provide efficient esterase-mediated activation, and exert antiplatelet effects through multiple mechanisms. While in vivo and clinical validation are still required, these findings highlight coumarins as a promising scaffold for developing multitarget cardiovascular therapeutics with improved pharmacokinetic profiles and reduced systemic toxicity.

### 2.5. Emerging and Multitarget Approaches

#### 2.5.1. Background and Rationale

Beyond specific disease categories, coumarin-based prodrugs are increasingly being explored as enabling tools for multitargeting and precision-controlled activation, particularly in pathological contexts characterized by network-level dysregulation. One emerging approach involves the design of coumarin-based hybrid prodrugs, in which the coumarin scaffold links or integrates multiple pharmacologically active motifs, enabling the concurrent modulation of inflammatory signaling, oxidative stress, and proliferative or microbial pathways [58]. These dual- or triple-action systems have demonstrated enhanced efficacy and reduced resistance in cancer and chronic inflammatory models, where monofunctional agents often fail to achieve durable responses [59,60]. A second rapidly developing strategy relies on the use of coumarins as stimulus-responsive properties, functioning as molecular switches that enable spatiotemporally controlled drug activation [61]. Photocleavable coumarin linkers have been widely employed to cage cytotoxic and neuroactive agents, allowing light-triggered release with high precision, while redox-responsive coumarin conjugates elevated reactive oxygen species levels typical of tumor and inflammatory microenvironments [62,63]. In this context, coumarins’ intrinsic fluorescence also enables theranostic applications in which drug delivery, activation, and biodistribution can be monitored in real time. These concepts are further extended through the interaction of coumarins into multifunctional nano-carrier systems, including enzyme-responsive nanoparticles and polymer–drug conjugates. In such architectures, coumarin motifs act simultaneously as cleavable linkers, targeting or activation reporters, and imaging probes, enabling unprecedented spatial and temporal control over drug release.

**Figure 7:**
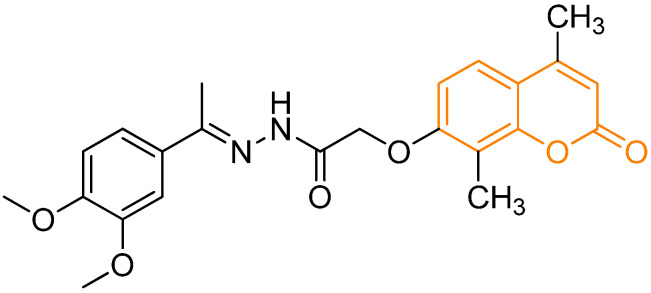
Molecular structure of the prodrug (*E*)-*N*’-(1-(3,4-dimethoxyphenyl)ethylidene)-2-((4,8-dimethyl-2-oxo-2*H*-chromen-7-yl)oxy)acetohydrazide, a coumarin-based analog combined with a curcumin derivative. In orange: the coumarinic moiety. Based on and adapted from Ghany et al., 2023 [64].

Another major strategy in this area involves hybrids that combine a coumarin core with other pharmacophores to enhance potency and achieve multimodal regulation of inflammation. A representative example is a series of curcumin-inspired coumarin hybrids designed to inhibit NO production and regulate cytokine release. They act by suppressing IL-6, IL-1β, and TNF-α. Mechanistic evaluation revealed modulation of AKT/mTOR and activation of the Nrf2/HO-1 antioxidant pathway, alongside inhibition of NF-κB nuclear translocation, demonstrating that coumarin derivatives can simultaneously act on oxidative stress and inflammatory cascades (Figure 7) [64].

#### 2.5.2. Global Conclusion on Multitarget Applications

Collectively, these emerging multitarget approaches position coumarin-based prodrugs not merely as delivery vehicles but as active, multifunctional components of next-generation therapeutic platforms aligned with the principles of polypharmacology and precision medicine demands.

The evidence presented highlights the remarkable versatility of coumarin-based prodrugs as multifunctional therapeutic platforms. Nonetheless, translating these multifunctional systems into clinical practice remains challenging. Increased molecular complexity can impact synthesis, stability, pharmacokinetics, and safety. Concurrent engagement of multiple mechanisms, although therapeutically advantageous, may also produce unintended off-target effects or synergistic toxicity. Overall, coumarin-based prodrugs exemplify the potential of next-generation polypharmacological platforms aligned with precision medicine principles. Future research should focus on robust in vivo validation, biocompatible nano-carrier integration, and careful assessment of combinatorial effects to ensure that the multifunctional promise of coumarins translates into safe and effective clinical therapies.

### 2.6. Summary of Coumarin-Based Prodrugs Across Therapeutic Areas and Considerations on Their Biotransformation

#### 2.6.1. Summary of Coumarin-Based Prodrugs Across Therapeutic Areas

To provide a concise overview of the diverse applications of coumarin-based prodrugs, Table 1 summarizes representative examples across major disease categories. The table highlights the specific prodrugs, their proposed mechanisms of activation, and the experimental models used to evaluate their efficacy. This structured summary illustrates the versatility of coumarin scaffolds as functional platforms for targeted drug delivery, controlled activation, and theranostic applications.

#### 2.6.2. Considerations on the Biotransformation via CYP 450 of Coumarin-Based Prodrugs

An important aspect that warrants deeper consideration is the metabolic fate and safety profile of coumarin-based prodrugs. Coumarin itself is primarily metabolized in humans by cytochrome P450 2A6 (CYP2A6), which converts coumarin into 7-hydroxycoumarin through 7-hydroxylation. However, CYP2A6 exhibits significant genetic polymorphism, leading to marked interindividual variability in metabolic capacity. Poor metabolizers may display slower clearance and prolonged systemic exposure to coumarin-derived fragments, whereas ultra-rapid metabolizers may exhibit accelerated activation or elimination kinetics. Such variability could directly impact both the predictability of prodrug activation and the safety margin of coumarin-based systems.

Furthermore, species differences in coumarin metabolism complicate translational extrapolation. While humans predominantly form 7-hydroxycoumarin, rodents generate higher proportions of hepatotoxic metabolites such as O-hydroxyphenylacetaldehyde via alternative oxidative pathways. Consequently, activation kinetics and toxicity profiles observed in murine models may not accurately predict human responses, underscoring the need for careful interspecies metabolic comparisons during preclinical development.

Although CYP2A6 is the primary enzyme responsible for coumarin 7-hydroxylation in humans, other cytochrome P450 isoforms may contribute to its metabolic fate under specific conditions. Minor roles have been attributed to CYP1A2 and CYP2E1, particularly at higher substrate concentrations or in individuals with reduced CYP2A6 activity. In addition, CYP3A4 may participate in the metabolism of structurally modified coumarin derivatives, especially in complex prodrug systems where steric factors alter enzyme accessibility. Importantly, alternative oxidative pathways can lead to the formation of coumarin 3,4-epoxide and downstream metabolites such as O-hydroxyphenylacetaldehyde, which have been associated with hepatotoxicity in certain species. Therefore, while CYP2A6 remains the dominant determinant of activation kinetics in humans, the potential contribution of additional CYP isoforms should be considered during prodrug optimization and safety assessment, particularly in populations with variable enzymatic expression or in the context of polypharmacy.

Long-term safety also requires consideration, particularly in systems designed for repeated or chronic administration. Although coumarin is generally regarded as having low toxicity at controlled doses, cumulative exposure to released coumarin fragments or reactive intermediates may pose risks of hepatotoxicity, enzyme induction, or drug–drug interactions. Repeated activation in inflamed or tumor microenvironments could further generate localized oxidative stress or metabolic burden. Therefore, comprehensive pharmacokinetic, toxicological, and metabolomic studies are essential to evaluate chronic exposure scenarios, especially in multifunctional or slow-release formulations.

## 3. Discussion

Coumarin-based compounds have long been recognized for their structural versatility and broad spectrum of biological activities. Historically, 4-hydroxycoumarin derivatives, such as warfarin and acenocoumarol, demonstrated the clinical potential of this scaffold in anticoagulation. However, despite decades of research, the direct clinical translation of most coumarin derivatives has remained limited, highlighting an intrinsic challenge: the scaffold itself often lacks sufficient potency or selectivity to serve as a standalone therapeutic agent. This limitation has shifted the focus toward the use of coumarins as functional scaffolds or promoieties for prodrug design, leveraging their predictable chemical reactivity, modularity, and potential for stimulus-responsive activation.

Importantly, the literature reveals that coumarin derivatives may play several distinct roles in drug design. In classical prodrug strategies, the coumarin scaffold primarily acts as a promoiety that enables stimulus-responsive drug release, often combined with fluorescent reporting of the activation process. In mutual prodrug systems, both the coumarin fragment and the conjugated drug contribute to biological activity following cleavage. Finally, in hybrid molecules, the coumarin moiety functions as a pharmacophoric element integrated into multifunctional structures designed to target multiple biological pathways simultaneously. Clearly distinguishing these different design strategies is essential to better understand the pharmacological potential and translational challenges associated with coumarin-based systems.

Across therapeutic areas, coumarin-based prodrugs exemplify the dual advantage of improving pharmacokinetic properties while enabling targeted or controlled drug release. In oncology, coumarins serve primarily as delivery vectors and theranostic agents, allowing tumor-selective activation via enzymatic, pH, or photoresponsive triggers. Similar strategies in inflammatory, infectious, and cardiovascular diseases demonstrate the scaffold’s ability to enhance oral bioavailability, reduce off-target toxicity, and enable site-specific drug activation.

The intrinsic fluorescence of many coumarin derivatives has further stimulated interest in their use as theranostic platforms. Fluorescent coumarin moieties allow real-time monitoring of drug activation, intracellular localization, and enzymatic cleavage processes in cellular systems. This property is particularly valuable for mechanistic studies, enabling the direct visualization of prodrug activation pathways and facilitating the optimization of linker design and activation kinetics. However, the excitation and emission wavelengths of most coumarin fluorophores generally lie in the visible region of the spectrum, which limits tissue penetration and therefore restricts their applicability for deep-tissue imaging in clinical settings. As a consequence, although coumarin fluorescence represents a powerful tool for mechanistic studies and preclinical validation, it remains less suitable for clinical imaging compared with established near-infrared probes or nuclear imaging modalities that allow deeper tissue penetration and quantitative detection in vivo.

Despite the conceptual advantages and abundant preclinical evidence, several critical challenges remain for clinical translation of coumarin-based prodrugs. Many of these systems combine multiple functional elements, including targeting groups, stimulus-responsive linkers, and imaging capabilities. While such multifunctional architectures provide elegant solutions at the molecular level, they can also introduce significant challenges related to synthetic complexity, scalability, stability, and regulatory evaluation. Moreover, the majority of reported studies remain confined to in vitro experiments or small-animal models, and comprehensive investigations of pharmacokinetics, biodistribution, metabolic stability, and long-term toxicity are still limited. Another important consideration is the multifunctional nature of emerging coumarin hybrids, which may combine drug delivery, pharmacological activity, and diagnostic properties within the same molecular framework. Although such multitarget systems align well with current trends in polypharmacology, they may also increase the risk of unexpected pharmacodynamic interactions or synergistic toxicity that could complicate their clinical development.

Nevertheless, the growing body of research underscores the strategic value of coumarins in next-generation prodrug design. Their chemical adaptability, intrinsic fluorescence, and biocompatibility make them ideal candidates for theranostic applications, multitarget interventions, and stimulus-responsive drug delivery. Future efforts should prioritize rigorous in vivo evaluation, scalable synthetic strategies, and careful assessment of combinatorial mechanisms to ensure that the multifunctional potential of coumarins translates into safe, effective, and clinically viable therapies. Ultimately, coumarin-based prodrugs illustrate a paradigm shift in medicinal chemistry: from active pharmacophores toward versatile molecular platforms capable of integrating delivery, targeting, and monitoring in a single scaffold.

While coumarin-based prodrugs represent an elegant and intellectually appealing strategy in medicinal chemistry, their greatest value may currently lie in their role as powerful mechanistic and design tools for preclinical research rather than as clinically translatable drug systems.

## 4. Conclusions

Coumarin-based compounds have demonstrated remarkable versatility as scaffolds for prodrug design across multiple therapeutic areas, including oncology, inflammation, infectious diseases, and cardiovascular disorders. While their intrinsic pharmacological activity is often limited, coumarins provide a chemically adaptable platform for improving drug solubility, stability, bioavailability, and targeted activation. The preclinical evidence reviewed highlights several key advantages of coumarin-based prodrugs: stimulus-responsive activation, including enzymatic-, pH-, redox-, and photo-triggered release, enabling precise spatial and temporal control over drug delivery; theranostic capabilities, leveraging intrinsic fluorescence for real-time monitoring of drug distribution, activation, and subcellular targeting; and multifunctionality and multitarget potential, allowing integration of therapeutic, diagnostic, and targeting functions within a single molecular framework. Despite these promising attributes, translation to clinical application remains challenging. Most studies are limited to in vitro or small-animal models, and the molecular complexity of advanced coumarin conjugates may present obstacles in synthesis, stability, and safety. Future research should focus on rigorous in vivo validation, scalable synthetic strategies, and careful evaluation of pharmacokinetics, biodistribution, and potential off-target effects.

Overall, coumarin-based prodrugs exemplify a shift from traditional pharmacologically active compounds toward versatile molecular platforms capable of integrating delivery, targeting, and monitoring functionalities. Their continued development holds significant potential for creating next-generation therapies that are safer, more selective, and aligned with the principles of precision medicine.

## 5. Materials and Methods

### 5.1. Search Strategy and Study Selection

A comprehensive search process was conducted on the following scientific databases to identify relevant studies published between 1 January 2010 and 19 February 2026: PubMed, Google Scholar and Web of Science. The primary search strategy combined the keyword “coumarin” with “prodrugs” and specific therapeutic areas, including: “cancer,” “inflammatory diseases and pain,” “infectious diseases,” “cardiovascular diseases,” “metabolic and liver diseases,” “neurodegenerative diseases,” and “emerging and multitarget approaches”. Additional targeted searches were performed using title-restricted queries (e.g., intitle: coumarin intitle: prodrugs cancer) to refine the selection and ensure retrieval of highly relevant articles specifically focused on coumarin-based prodrug strategies. Boolean operators (AND) were systematically applied to combine keywords and optimize specificity. Reference lists of selected articles were also manually screened to identify additional relevant publications not captured during the primary database search. To summarize, the main search was conducted using the keywords: ‘Coumarin’ AND ’cancer’ AND ’prodrugs’; ‘Coumarin’ AND ’inflammatory diseases and pain’ AND ’prodrugs’; ‘Coumarin’ AND ’infectious diseases’ AND ’prodrugs’; ‘Coumarin’ AND ’cardiovascular diseases’ AND ’prodrugs’; ‘Coumarin’ AND ’metabolic and liver diseases’ AND ’prodrugs’; ‘Coumarin’ AND ’neurodegenerative diseases’ AND ’prodrugs’; ‘Coumarin’ AND ‘emerging and multitarget approaches’ AND ’prodrugs’; ‘intitle: coumarin intitle: prodrugs cancer’; ‘intitle: coumarin intitle: prodrugs inflammatory diseases and pain’; ‘intitle: coumarin intitle: prodrugs infectious diseases’; ‘intitle: coumarin intitle: prodrugs cardiovascular diseases’; ‘intitle: coumarin intitle: prodrugs metabolic and liver diseases’; ‘intitle: coumarin intitle: prodrugs neurodegenerative diseases’; ‘intitle: Coumarin intitle: prodrugs emerging and multitarget approaches’.

### 5.2. Eligibility Criteria and Data Extraction

All retrieved references were independently screened and categorized according to study type, including in vitro biological evaluations, in vivo preclinical investigations, and clinical studies. Original research articles describing the design, synthesis, biological evaluation, or therapeutic application of coumarin-based prodrugs were prioritized. Review articles were also included to provide contextual background for each pathological area and to ensure a comprehensive understanding of the current state of knowledge. Publications not directly related to coumarin-based prodrug strategies, as well as duplicate records, were excluded.

## Figures and Tables

**Table 1:** Representative Coumarin-Based Prodrugs: Mechanisms and Experimental Models.

Disease/Condition and Advantages of Prodrug Development	Coumarin-Based Prodrug	Mechanism/Activation	Development Stage
Cancer Targeted release of cytotoxic molecules via enzymatic or redox activation Intrinsic antitumor effect of coumarin Fluorescence for intracellular tracking	5-FU–coumarin conjugates	Esterase-triggered cleavage; tumor-selective release	In vitro
	pH-responsive doxorubicin– coumarin conjugates	Acidic tumor microenvironment triggers linker hydrolysis	In vitro
	Artemisinin–coumarin conjugates	Mitochondrial targeting; ROS-mediated cytotoxicity	In vitro
	Gemcitabine–coumarin–biotin conjugates	Glutathione-sensitive disulfide cleavage; biotin-mediated uptake	In vitro
	Light-activated coumarin prodrugs (e.g., combretastatin A-4 conjugates)	Photo-triggered cleavage; spatiotemporal control	In vitro
	DEACAS-coumarin lipid nanocapsules	Red-light photoactivation of masked melphalan	In vivo (preclinical)
	Two-photon activatable photosensitizer with coumarin	Sulfite-triggered activation; tumor ablation	In vivo (preclinical)
	DHA–coumarin nanosystem	Mitochondrial targeting; fluorescent monitoring	In vitro/In vivo (preclinical)
Inflammation/Pain Masking of NSAIDs to reduce gastrointestinal toxicity Localized enzymatic release Intrinsic anti-inflammatory activity	Flurbiprofen–umbelliferone	Esterase cleavage; reduces gastric toxicity	In vivo (preclinical)
	5-ASA–7-AMC (azo-coumarin)	Colon-targeted bacterial azoreductase cleavage; fluorescence activation	Ex vivo/In vitro
	*N*-alkylated γ-lactone derivatives of AI-77-B	Oral absorption; NADPH-dependent enzymatic N-dealkylation	In vivo (preclinical)
Infectious Diseases-Antibacterial Targeted activation by bacterial or fungal enzymes Coumarin may have intrinsic antimicrobial activity Monitoring of cleavage and cellular uptake through fluorescence	Glycosylated coumarins (e.g., Esculin)	β-Glucosidase-mediated hydrolysis to active esculetin	In vitro
	α-Carbonic anhydrase-targeting coumarins	Suicide inhibition by enzyme-mediated lactone hydrolysis	In vitro
	Coumarin–benzimidazole conjugates	Inhibition of viral replication	In vitro/In vivo (preclinical)
Cardiovascular Improved oral bioavailability of peptidomimetics (e.g., RGD analogs) Controlled release of anticoagulants or antithrombotic agents Antioxidant and vasoprotective effect	Cyclic coumarin prodrugs of RGD peptidomimetics	Esterase-mediated activation; enhanced oral absorption	In vitro/In vivo (preclinical)
	Coumarin, esculetine, esculine	Platelet aggregation inhibition; COX-1 modulation	In vitro
Emerging/Multitarget Versatile chemical platform Enabling of multi-target design and mechanistic tracking	Curcumin-inspired coumarin hybrids	Suppression of IL-6, IL-1β, TNF-α; modulation of AKT/mTOR, Nrf2/HO-1, NF-κB	In vitro/In vivo (preclinical)

## Data Availability

No new data were created or analyzed in this study.

## Abbreviations

The following abbreviations are used in this manuscript:

5-FU	5-fluorouracil
AMC	7-amino-methylcoumarin
ASA	Aminosalicylic acid
AZT	Azidothymidine
BBB	Blood–brain barrier
CA-4	Combretastatin A-4
COX	Cyclooxygenase
CYP	Cytochromes P450
DHA	Dihydroartemisinin
IC_50_	Half maximal inhibitory concentration
MIC	Minimal inhibitory concentration
NO	Nitric oxide
NSAIDs	Nonsteroidal anti-inflammatory drugs
PDT	Photodynamic therapy
ROS	Reactive oxygen species
t_1/2_	Half-life
